# The Die Is Cast: Decision‐Making Under Risk and Under Ambiguity in Schizophrenia and Alcohol Use Disorder

**DOI:** 10.1002/cpp.70178

**Published:** 2025-11-24

**Authors:** Sarah Stumpp, Alexander Wolber, Natascha Büchele, Leonie Lipinski, Stephanie N. L. Schmidt, Brigitte Rockstroh, Michael Odenwald, Daniela Mier

**Affiliations:** ^1^ Department of Psychology University of Konstanz Constance Germany

**Keywords:** cognition, decision‐making, Game of Dice Task, Iowa Gambling Task, psychosis

## Abstract

Patients with schizophrenia (SZ) tend towards riskier decision‐making (DM). Yet the specificity of these findings, as well as the impact of impairments in executive functions (EF), has not been sufficiently clarified. In a preregistered study (https://osf.io/n7z6y) 40 SZ, 50 patients with alcohol use disorder (AUD) and 36 healthy controls (HC) completed an n‐back task (as EF challenge), the Game of Dice Task (GDT; DM under risk) and the Iowa Gambling Task (IGT; DM under ambiguity). AUD and SZ performed worse than HC in the n‐back task and riskier in the GDT. In the IGT, only AUD performed riskier than HC and preferred disadvantageous Deck B compared to SZ. However, controlling for demographics and IQ abolished significance. Correlations of performance in the GDT and IGT with working memory differed between groups. Taken together, both patient groups show a deficit in the reflective system, whereas only AUD show impairments in the impulsive system. Thus, the direct comparison of DM in SZ and AUD reveals a differential DM profile of SZ and AUD. Our results suggest that both groups may benefit from EF training, especially in planning, categorization and cognitive flexibility, whereas AUD could additionally profit from impulse control and inhibition training. However, the findings need replication with well‐matched samples, and the link between EF and DM in SZ should be examined more carefully with experimental approaches.

## Summary

- Decision‐making impairments are present in both patients with schizophrenia (SZ) and patients with alcohol use disorder (AUD) but differ in nature, with SZ showing deficits primarily in reflective processing and AUD additionally displaying impulsive decision‐making patterns.
- Executive functions (EF), particularly working memory, play a key role in these decision‐making deficits, highlighting the importance of cognitive assessments in clinical evaluations.
- EF training may be beneficial for both SZ and AUD, especially targeting planning, categorization and cognitive flexibility; AUD may also require interventions focused on impulse control and inhibition.

## 1 Introduction

Patients with schizophrenia (SZ) tend towards riskier decisions; that is, they choose riskier options and make more disadvantageous decisions (Betz et al. 2019; Woodrow et al. 2019). Yet it remains unclear whether SZ exhibit dysfunctional decision‐making (DM) under ambiguity and under risk, how specific these deficits are for SZ and how impaired executive functions (EF) impact DM in SZ.

Decision‐making under ambiguity means that outcomes are not predictable, and the decision has to be made without or with only little information about the probabilities for reward and punishment. Under such conditions, feedback from previous decisions serves to identify advantageous alternatives (Bechara and Damasio 2005). In contrast, DM under risk is defined as decisions on the basis of explicit information about the conditions and the associated consequences (Brand et al. 2005); hence, predictable outcomes are defined. Behavioural and cortical correlates of these two facets of DM have been examined using the Iowa Gambling Task (IGT; Bechara et al. 1994, 2000) and the Game of Dice Task (GDT; Brand et al. 2005). The IGT probes DM under ambiguity as implicit rules for gains and losses must be figured out from each decision outcome (i.e., feedback; Bechara et al. 1994; Bechara et al. 2000). It was initially developed to confirm the somatic marker theory, which states that emotions play a crucial role in DM by generating behaviourally relevant bodily signals based on past experiences (Damasio 1996). Brand, Recknor, et al. (2007) suggest that the IGT shifts from ambiguity to risk because an association between IGT performance and EF is found only in the last trials of the task. Designed as a gambling task, the GDT targets the effect of EF on DM under risk with explicit rules for winning and losing (Brand et al. 2005). IGT and GDT are supposed to activate specific neuropsychological systems that mediate DM: the impulsive system and the reflective system (Bechara 2005; Schiebener and Brand 2015). According to Bechara (2005), the impulsive system is driven by learned emotional evaluations of situations, whereas the reflective system is characterized by cognitive reasoning. However, both systems may interact during DM. Colautti et al. (2022) suggest in their review that different types of EF are required for completing the IGT and GDT. Although IGT performance is associated with so‐called hot EF that are related to emotion regulation and reward processing, GDT performance is linked to cold EF, such as logical reasoning. This is in agreement with the assumption of an involvement of the impulsive system in DM under ambiguity and of the reflective system in DM under risk (Bechara 2005; Colautti et al. 2022).

The current literature offers no clear priority of either system abnormality in SZ: Deficits have been reported in the IGT (e.g., Betz et al. 2019; Woodrow et al. 2019) and GDT (e.g., Li et al. 2021; Zhang et al. 2015) but remain inconclusive regarding the predominant dysfunctional system (Fond et al. 2013; Lee et al. 2007; Li et al. 2021; Pedersen et al. 2017; Zhang et al. 2015). Studies that applied both, the IGT and the GDT, either showed impairments in both tasks (Fond et al. 2013; Zhang et al. 2015), in the IGT, but not in the GDT (Lee et al. 2007), or stronger impairments in the GDT than in the IGT (Li et al. 2021; Pedersen et al. 2017). Importantly, Fond et al. (2013), Li et al. (2021) and Lee et al. (2007) reported deficits in the IGT only in the last blocks, which supports a deficit in DM under risk and not under ambiguity ‐ also in the IGT (Brand, Recknor, et al. 2007). Given that impaired feedback processing and dysfunctional EF are common in SZ, that is, evidence for disturbances in the impulsive and in the reflective system (Forbes et al. 2009; Gold et al. 2008; Lett et al. 2014; Waltz et al. 2013), the present study compared GDT and IGT performance and an n‐back task as EF challenge in a sample of SZ to differentiate the causes of risky DM in SZ.

There might be several factors that influence the functioning of the impulsive and reflective system during DM in SZ. Regarding the association between risky DM and psychosis symptomatology, a review by Purcell et al. (2022) showed inconsistent evidence. For example, Pedersen et al. (2017) found that positive symptoms were related to riskier decisions in the GDT. Fond et al. (2013), Li et al. (2021) and Lee et al. (2007), however, did not find significant associations between IGT or GDT performance and psychosis symptomatology. Further, Beninger et al. (2003) found impairments in the IGT in patients with atypical antipsychotic medication but not in those with typical antipsychotics. However, more recent studies showed no significant associations between antipsychotic medication and DM in SZ (Fond et al. 2013; Pedersen et al. 2017; Struglia et al. 2011). Moreover, Toplak et al. (2010) reported an association between IGT performance and IQ in SZ, but to our knowledge, there are no studies on the association of GDT and IQ in SZ. Given the heterogeneous and sparse findings, we aimed to reexamine these associations in our sample.

Another open question is whether and if yes, which DM impairments are specific for SZ. In particular, patients with alcohol use disorder (AUD) seem to have similar deficits in DM as SZ. The aforementioned neurobiological model by Bechara (2005) was initially proposed for AUD and shows interesting similarities to the dopamine hypothesis model by Howes and Kapur (2009) for SZ. In both models, deficits in the dopamine system, as well as aberrations in associated limbic regions and in the prefrontal cortex, are emphasized. Further, fMRI studies show aberrations in the connectivity of striatal regions and prefrontal cortex in both SZ (Sarpal et al. 2015) and AUD (Becker et al. 2017). In AUD, studies have repeatedly confirmed a tendency towards risky DM (e.g., review by Ariesen et al. 2023). Poor IGT and GDT performance of AUD compared to healthy controls (HC) has been frequently reported (Kim et al. 2011; Kovács et al. 2017; Xie et al. 2018), importantly in the last IGT blocks (see Ariesen et al. 2023). Thus, the EF deficits that are found in SZ and AUD (Stephan et al. 2017; Thai et al. 2019) could be related to impaired DM under risk, especially in the last blocks of the IGT. As the evidence in AUD consistently points to risky DM, this group is a suitable clinical control group for SZ to investigate the specificity of DM impairments.

To summarize, studies on impaired DM in SZ show inconsistent results, and there are only a few studies that have examined both IGT and GDT in SZ (Fond et al. 2013; Lee et al. 2007; Li et al. 2021; Pedersen et al. 2017), and to our knowledge, there are currently no studies that have directly compared DM in the IGT and GDT in AUD and SZ. The present study compared SZ and AUD and a sample of HC to investigate the nature of the DM deficit in SZ and to clarify the impact of EF on DM. Because the current body of research on SZ is heterogeneous, no hypotheses were preregistered. Using an exploratory approach, we aimed (1) to compare the performance of SZ in DM under ambiguity and under risk with AUD and HC (2) and to assess the influence of EF on DM in SZ and AUD. In addition, (3) we intended to explore the association between IGT and GDT performance and psychosis symptomatology and (4) to analyse the impact of antipsychotic medication and IQ on DM.

## 2 Method

The present data are part of a larger preregistered project (https://osf.io/n7z6y) on risk perception and behaviour in SZ and AUD, which encompassed several sessions. The description of the complete project and further results will be reported elsewhere. Regarding the IGT and GDT, no hypotheses were preregistered. The project was approved by the ethics board of the University of Konstanz.

### 2.1 Participants

We recruited adult inpatients diagnosed with schizophrenia spectrum disorder, inpatients diagnosed with alcohol dependence, and HC. A total of 155 participants were enrolled in the study. Exclusion criteria for all participants were (a) age < 18 years; (b) insufficient command of the German language; (c) lifetime diagnosis of neurological disorders; (d) an acute psychotic episode, acute suicidality or not being distanced from self‐harming behaviours; and (e) for AUD a diagnosis of drug dependence (except nicotine and caffeine), for SZ a diagnosis of alcohol or drug dependence (except nicotine and caffeine), and HC were excluded if they reported lifetime psychiatric hospital treatments or any mental disorders (except specific phobia and adjustment disorder, nicotine and caffeine dependence) within the last 5 years.

*N* = 49 schizophrenia inpatients were recruited from the psychosis wards of the Center of Psychiatry Reichenau (ZfP), Germany, with a primary F2 diagnosis according to the International Classification of Diseases Version 10 (ICD‐10; for German translation Dilling et al. 2015). Because of dropout between the first and second appointment (*n* = 8) and aggravation of psychotic symptoms in one participant, the final SZ sample consisted of 40 patients with primary diagnosis F20 (*n* = 23), F22 (*n* = 2), F23 (*n* = 6), F25 (*n* = 8) and F28 (*n* = 1). All SZ patients were under antipsychotic medication (Table 1). The antipsychotic dose equivalents were calculated according to Gardner et al. (2010) and Leucht et al. (2016) using the software chlorpromazineR 0.2.0 (http://eebc.ca/chlorpromazineR_shiny/). PANSS data of one patient are not available (Table 1).

**TABLE 1:** Demographic and clinical information of patients and healthy controls (mean [SD]).

	SZ *n* = 40	AUD *n* = 50	HC *n* = 36	Statistics
Gender (female:male)	9:31	13:37	16:20	*χ* ^2^ (2) = 5.012, *p* = 0.082
Age (years)	33.15 (11.46)	44.80 (11.23)	33.56 (14.65)	*F*(2, 123) = 12.954, *p* < 0.001
Education (years)	10.87 (1.61)^a^	10.48 (1.22)	11.33 (0.96)	*F*(2, 77.01) = 6.593, *p* = 0.002
Verbal IQ	101. (11.9)	98.64 (9.12)	103.47 (11.44)	*F*(2, 123) = 2.272, *p* = 0.107
CPZ equivalents	453.99 (267.11)	87.50 (96.59)		*t*(41.395) = 7.032, *p* < 0.001
PANSS^a^				
Pos	12.08 (4.68)			
Neg	13.35 (5.22)			
General	27.21 (6.51)			
Total	52.64 (12.32)			

*Note:* Within the AUD group, antipsychotic medications were administered to 10 patients.

Abbreviations: CPZ equivalents, chlorpromazine equivalent in mg per day; PANSS, Positive and Negative Syndrome Scale; Pos, PANSS positive scale with a range from 7 to 24 in our sample; Neg, PANSS negative scale with a range from 7 to 25 in our sample; General, PANSS general psychopathology with a range from 18 to 41 in our sample; Total, PANSS total score with a range from 33 to 79 in our sample.

^a^ *n* = 39.

*N* = 61 AUD inpatients from the addiction wards (primary diagnosis F10.2) of the ZfP were recruited. Because of dropout between the first and second appointment (*n* = 9) and technical problems during the measurement (*n* = 2), the final AUD sample consisted of 50 patients (Table 1). Comorbid diagnoses in both samples were harmful use of substances (SZ: *n* = 6, AUD: *n* = 10); affective disorders (SZ: *n* = 6, AUD: *n* = 28); neurotic, stress‐related and somatoform disorders (SZ: *n* = 7, AUD: *n* = 12); bulimia nervosa (AUD: *n* = 1); personality disorders (SZ: *n* = 6, AUD: *n* = 10); and ADHD/ADD (SZ: *n* = 3, AUD: *n* = 6).

HC (*N* = 39) were recruited via public notices advertising flyers distributed in various public institutions. Because of technical problems during the appointment, the final sample consisted of 36 participants.

Despite our efforts to match for age, gender, IQ and education, groups differed significantly (Table 1). Post hoc Tukey‐HSD tests revealed significant differences in the mean age between SZ and AUD (*p* < 0.001), as well as between AUD and HC (*p* < 0.001) and between AUD and HC (*p* = 0.001) in the education level.

### 2.2 Procedure and Design

HC were informed about the aims and the procedure in a telephone interview, in which trained study staff completed a screening for eligibility. Patients were first identified as potential participants by their psychologists/psychiatrists and afterwards recruited by the study staff.

The first appointment started with information about the study aims and procedures. Participants signed written informed consent, followed by the German version of the Structured Clinical Interview for DSM‐5 Disorders–Clinical Version (SCID‐CV; Beesdo‐Baum et al. 2019a). The SZ sample additionally received the German translation of the Structured Clinical Interview for the Positive and Negative Syndrome Scale (PANSS; Kay et al. 1987). All participants filled in several questionnaires, including the German version of the Structured Clinical Interview for DSM‐5 Disorders–Screening Personality Questionnaire (SCID‐SPQ; Beesdo‐Baum et al. 2019c).

For patients who fulfilled the number of criteria for a given diagnosis in the SCID‐SPQ, the second appointment started with the German version of the Structured Clinical Interview for DSM‐5 Disorders–Personality Disorders (SCID‐PD; Beesdo‐Baum et al. 2019b). After a practice round of the n‐back task, all participants started the main session with the n‐back task, followed by the GDT and the IGT. Subsequently, verbal IQ was assessed with the Wortschatztest (WST, Schmidt and Metzler 1992), and participants completed further questionnaires. Both sessions lasted around 2 h and were compensated with 25 euros each, directly after participation, regardless of whether the participants won or lost fictitious money in the tasks.

The data were collected between July 2021 and September 2023, partially during the COVID‐19 pandemic. Therefore, participants had to adhere to local regulations, including wearing face masks and testing negative for COVID‐19.

### 2.3 Measures and Instruments

#### 2.3.1 n‐Back Task

We assessed working memory with a computerized version of the n‐back task with a 2‐back experimental and a 0‐back control condition (Callicott et al. 1999). Participants were instructed and completed several training trials. The numbers 1–4 were shown on a diamond‐shaped set‐up on the screen. In the 0‐back condition (control condition), participants had to press the button according to the currently displayed number. In the 2‐back condition (working memory condition), participants had to press the number that was presented two trials before. The response pad had four different buttons, which represented the corresponding numbers. Participants were instructed only to press a button when a stimulus was presented. Early and late pressing was automatically considered as an error. Each stimulus was presented for 500 ms, and the time between two stimuli was 1500 ms. The two conditions alternated within eight blocks in which the sequence of the numbers was randomized. A cue indicating the current condition was always displayed at the top of the screen. As a parameter for working memory, we calculated the percentage of correct responses in the 2‐back minus 0‐back condition.

#### 2.3.2 Game of Dice Task

**FIGURE 1:**
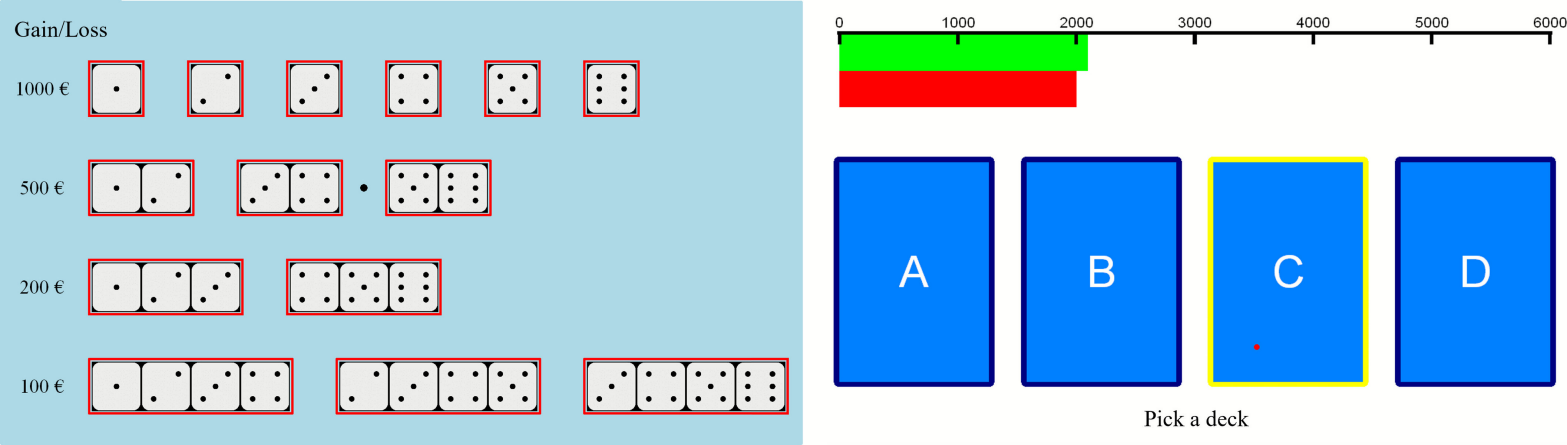
Design of the Game of Dice Task (left) and the Iowa Gambling Task (right).

For assessing DM under risk, the GDT (Brand et al. 2005) was applied. Instructions were provided orally with the help of a PowerPoint presentation for task visualization. Participants were asked to maximize their fictive starting capital of 1000 euros. For 18 trials in total, they should make a guess as to which number a single die will show, before each throw. They had to decide between 14 choices: one single number or a combination of two, three or four numbers with different monetary gains and losses (Figure 1). The gains/losses were constantly presented on the screen and were associated with the winning probabilities (Figure 1).

Each of the six possible numbers of spots on the die occurred three times in a pseudorandomized order. Decisions had to be made within 4 s, and after each decision, the gain/loss and the adjusted capital were presented on the screen. Choosing combinations of three or four numbers is advantageous because of the winning probabilities of 50% or higher.

First, we examined how often a single number, combinations of two, three or four numbers were chosen. Further, we analysed the number of risky (single and double numbers) and nonrisky (three and four numbers) choices and calculated a net score by subtracting risky from nonrisky choices. Higher net scores indicate nonrisky performance.

#### 2.3.3 Iowa Gambling Task

For assessing DM under ambiguity, participants were introduced to a computerized version of the IGT (Bechara et al. 1994; Bechara et al. 2000). Instructions were provided orally with the help of a PowerPoint presentation for task visualization. The IGT consists of four decks of cards, A, B, C, and D (Figure 1), each deck counting 40 cards. Participants were instructed that in each trial they should choose one card from one of the four decks. They were informed that they should maximize their profit and that some decks are worse than others. They started with a fictional credit of 2000 euros that was visualized and updated after each choice. After each decision, participants received feedback about their amount of gain and loss and the updated total amount. A total of 100 trials were conducted self‐paced within about 10 min. In our IGT, choosing cards from Decks A and B was disadvantageous and from Decks C and D advantageous.

We examined the frequency of each chosen deck, the number of disadvantageous (sum of choices of Decks A and B) and advantageous choices (sum of choices of Decks C and D) and calculated an IGT net score by subtracting the number of disadvantageous from the number of advantageous choices. Higher net scores indicate advantageous performance. For examination of potential changes in the DM strategies during the IGT, we conducted a blockwise analysis (5 blocks à 20 trials).

#### 2.3.4 Positive and Negative Syndrome Scale

Symptom severity was evaluated using the PANSS (Kay et al. 1987). The 30‐item measurement assesses positive and negative symptoms as well as general psychopathology. The responsible psychologist or psychiatrist rated the severity of each item over the past 7 days on a Likert scale ranging from 1 (*absent*) to 7 (*extreme*), based on information obtained from an interview with the patient and additional observations of the patient's daily ward routines.

### 2.4 Statistical Procedures and Handling of Missing Data

Statistical analyses were carried out with SPSS Version 29.0. Because of the sample size (*n* ≥ 30) in each of the three groups, parametric methods were used (Blanca et al. 2017; Schmider et al. 2010). There were no clear outliers in the data. For all analyses, the level of statistical significance was set at *p* < 0.05. The reported group differences in demographic data were analysed with *χ* ^2^ test, one‐way ANOVA and Welch tests. To assess group differences in the n‐back task, in the GDT and in the IGT, we conducted one‐way ANOVAs. Furthermore, we conducted a one‐way ANCOVA for assessing potential confounding effects of the three key demographic variables (gender, age and education) and IQ on the GDT net score between the groups and the same plus working memory on the IGT.

To compare differences in choice preference in the GDT, we conducted a repeated‐measures ANOVA (rmANOVA) with choice as within‐subject factor and group as between‐subject factor, and a rmANCOVA for assessing potential confounding effects of demographics and IQ on the selection of the choices in the GDT.

To investigate potential differences across the IGT in each group within the five blocks, we conducted a rmANOVA with block as the within‐subject factor and group as the between‐subject factor. Furthermore, we conducted a rmANCOVA for assessing potential confounding effects of demographics, IQ and working memory on the IGT net score.

Additionally, Pearson correlation analyses were used to examine the correlations between DM performances, n‐back performance, demographic and clinical variables (age and education, antipsychotic medication and symptom severity) and IQ within each group.

## 3 Results

### 3.1 Working Memory Performance in the n‐Back Task

As shown in Table 2, both clinical groups showed poorer performance in the n‐back task relative to HC. However, a specific deficit in SZ was not found.

**TABLE 2:** Performance per task and group.

	1. SZ *n* = 40	2. AUD *n* = 50	3. HC *n* = 36	*F*	*p*	Significant group differences^a^
n‐back						
% correct responses 0‐back	95.98	91.53	96.08	1.38^b^	0.257	
% correct responses 2‐back	52.19	49.50	68.87	7.15	0.001	1, 2 < 3
% correct responses 2‐back minus 0‐back	−43.79 (24.97)	−42.04 (25.36)	−27.22 (20.57)	5.51	0.005	1, 2 < 3
GDT						
Net score	5.95 (11.49)	6.16 (10.57)	11.39 (7.62)	4.76^b^	0.011	1, 2 < 3
Single number	2.05 (4.19)	1.72 (3.85)	0.81 (1.75)	1.27	0.284	
Two numbers	3.97 (4.53)	4.20 (4.23)	2.50 (2.48)	2.18	0.118	
Three numbers	5.57 (4.96)	6.56 (4.5)	8.72 (5.32)	4.09	0.019	1 < 3
Four numbers	6.4 (5.74)	5.52 (5.02)	5.97 (5.15)	0.31	0.735	
Total money (€)	−700.00 (2998.29)	−918.00 (3201.00)	375.00 (1562.85)	2.48	0.08	
IGT						
Net score	11.25 (25.33)	−0.52 (27.51)	13.17 (23.46)	3.72	0.027	2 < 3
Deck A	18.87 (7.12)	18.34 (7.78)	15.72 (7.34)	1.95	0.147	
Deck B	25.50 (9.46)	31.92 (9.75)	27.69 (9.32)	5.30	0.006	1 < 2
Deck C	27.80 (8.66)	21.32 (9.79)	25.00 (9.73)	5.34	0.006	2 < 1
Deck D	27.83 (8.97)	28.42 (10.05)	31.58 (8.87)	1.76	0.177	
Total money (€)	1891.88 (709.40)	1744.00 (819.93)	2049.31 (839.59)	1.56	0.214	

^a^ Pairwise comparisons, the mean difference is significant at the 0.05 level, Tukey post hoc tests for equal variances, Games–Howell tests for unequal variances.

^b^ Welch Test is reported because of the heterogeneity of variances.

### 3.2 DM in the GDT

Both groups showed impaired DM under risk. GDT net scores differed significantly between groups (partial *η* ^2^ = 0.055). Games–Howell post hoc analysis revealed significant differences between SZ and HC (*p* = 0.043, *d* = −0.552) and between AUD and HC (*p* = 0.025, *d* = −0.506). GDT net score did not differ significantly between the two clinical samples (Table 2). After controlling for demographic variables and IQ, significance was not reached *F*(2, 118) = 2.03, *p* = 0.136, partial *η* ^2^ = 0.033.

A rmANOVA revealed a main effect of choice, Greenhouse–Geisser (GG) *F*(2.59, 318.61) = 27.78, *p* < 0.001, partial *η* ^2^ = 0.184, and a trend for an interaction between choice and group, GG *F*(5.18, 318.61) = 1.95, *p* = 0.083, partial *η* ^2^ = 0.031, but no main effect of group (*p* = 1.00). Bonferroni‐adjusted post hoc analysis revealed significant differences between all choices (all *p*s < 0.05), except between the choices of three and four numbers (*p* = 1.00). Post hoc tests for group differences within the four different choices were only significant for three numbers (*p* = 0.016); all other comparisons were not significant (see Table 2).

When controlling for demographics and IQ as covariates, neither the main effects (choice: GG *F*[2.58, 304.83] = 0.80, *p* = 0.477, partial *η* ^2^ = 0.007; group: *F*[2, 118] = 0.000, *p* = 1.00, partial *η* ^2^ = 0.000) nor the interaction (GG *F*[5.17, 304.83] = 1.65, *p* = 0.145, partial η^2^ = 0.027) remained significant.

### 3.3 DM in the IGT

AUD, but not SZ, showed impaired DM under ambiguity. IGT net scores differed significantly between groups (Table 2). IGT net scores did not differ significantly between SZ and HC (*p* = 0.944), but between SZ and AUD as a trend (*p* = 0.083, *d* = 0.443), whereas AUD performed significantly poorer than HC (*p* = 0.043, *d* = −0.529). Yet, when controlling for demographics, IQ and working memory, the differences in the IGT net score between the three groups were no longer significant *F*(2, 117) = 1.599, *p* = 0.206, partial *η* ^2^ = 0.027.

**FIGURE 2:**
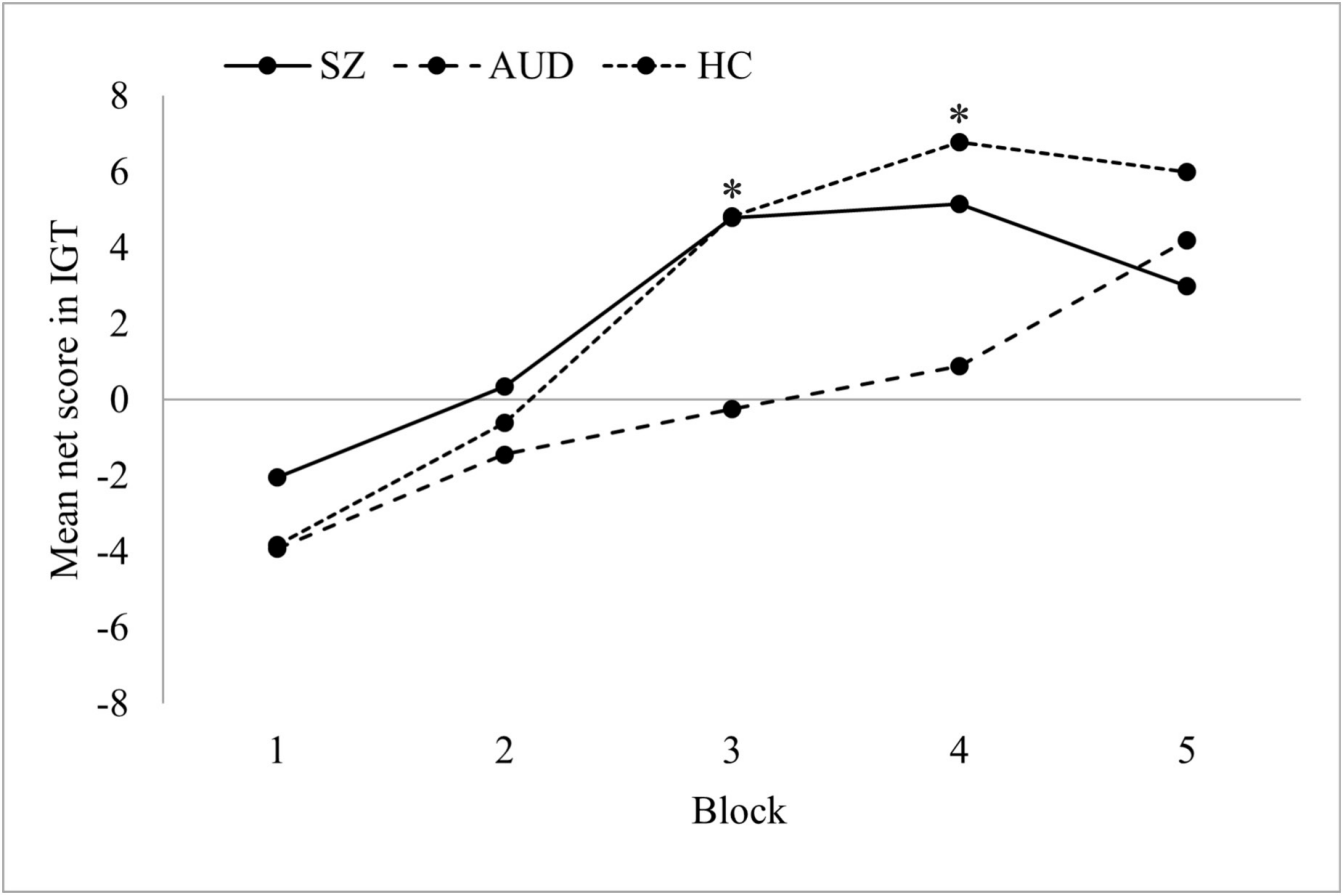
Mean net scores (advantageous minus disadvantageous choices) across the five blocks in the Iowa Gambling Task (IGT) for schizophrenia (SZ), alcohol use disorder (AUD) and healthy controls (HC). *p < 0.05.

The rmANOVA with block as a within‐subject factor and group as a between‐subject factor confirmed significant differences between the IGT net scores in the 5 blocks, GG *F*(3.07, 378.02) = 22.92, *p* < 0.001, partial *η* ^2^ = 0.16, and between the groups *F*(2, 123) = 3.72, *p* = 0.027, partial *η* ^2^ = 0.057 (see Figure 2). The interaction block × group approached significance, GG *F*(6.15, 378.02) = 1.91, *p* = 0.077, *η* ^2^ = 0.030.

The groups differed significantly in block 3 (*η* ^2^ = 0.075; see Figure 2). According to the post hoc analysis, SZ and HC scored higher than AUD (*p* = 0.021 and *p* = 0.025), and there was no significant difference between SZ and HC (*p* = 1.00). There were also significant differences between the groups in block 4 (*η* ^2^ = 0.073). According to the Games–Howell post hoc test, HC scored significantly higher than AUD (*p* = 0.009, *d* = 0.674), and there was a trend for higher scores in SZ compared to AUD (*p* = 0.09, *d* = 0.462). In block 5, although SZ exhibited a decline, mean comparisons did not reach statistical significance (*p* = 0.383). Descriptive differences were observed with marginal differences between HC and AUD and between AUD and SZ, whereas more pronounced differences were evident between SZ and HC (Figure 2). When controlling for demographics, IQ and working memory, neither the main effects (block: GG *F*[3.09, 361.22] = 0.65, *p* = 0.590, partial *η* ^2^ = 0.006; group: *F*[2, 117] = 1.6, *p* = 0.206, partial *η* ^2^ = 0.027) nor the interaction (GG *F*[6.18, 361.22] = 1.26, *p* = 0.275, partial *η* ^2^ = 0.021) remained significant.

### 3.4 Correlational Analyses

The correlational analyses showed differential associations for the groups (see Table 3).

**TABLE 3:** Pearson correlation coefficients per measurement, including CPZ equivalents and IQ, and group.

	SZ *n* = 40	AUD *n* = 50	HC *n* = 36
GDT net score & IGT net score	0.243	0.038	0.381*
GDT net score & n‐back	0.300^+^	0.001	0.594**
IGT net score & n‐back	0.435**	0.215	0.259
Verbal IQ & n‐back	−0.082	−0.081	0.190
Verbal IQ & GDT net score	−0.013	0.275*	0.488**
Verbal IQ & IGT net score	−0.214	0.175	−0.006
CPZ equivalents & n‐back	−0.137		
CPZ equivalents & GDT net score	−0.108		
CPZ equivalents & IGT net score	−0.003		
PANSS^a^ pos & n‐back	−0.283^+^		
PANSS^a^ neg & n‐back	0.114		
PANSS^a^ general & n‐back	−0.094		
PANSS^a^ pos & GDT net score	0.176		
PANSS^a^ neg & GDT net score	−0.208		
PANSS^a^ general & GDT net score	−0.138		
PANSS^a^ pos & IGT net score	−0.283^+^		
PANSS^a^ neg & IGT net score	0.260^+^		
PANSS^a^ general & IGT net score	−0.091		

Abbreviations: CPZ equivalents, chlorpromazine equivalent in mg per day; GDT, Game of Dice Task; IGT, Iowa Gambling Task; n‐back, percentage of correct responses in the 2‐back minus 0‐back condition; PANSS pos, PANSS positive scale; PANSS neg, PANSS negative scale; PANSS general, PANSS general psychopathology.

^a^ *n* = 39.

^+^ *p* < 0.1.

* *p* ≤ 0.05.

** *p* < 0.01.

## 4 Discussion

The established DM tasks, GDT and IGT, and the comparison with AUD and HC served to clarify the conditions (ambiguity or risk) that prompt dysfunctional, risky DM in SZ, and to explore the specificity of DM impairments in SZ. The additional comparison of n‐back performance between groups and correlation with DM performance tested the impact of EF on dysfunctional DM in SZ. Moreover, the association of symptom severity, antipsychotic medication and IQ with task performance in SZ was explored. Results confirm impaired DM in SZ and AUD under risk, in AUD under ambiguity, as well as deficits in EF in both SZ and AUD. They further indicate that impaired EF may influence DM under ambiguity risk in SZ. Clear conclusions, though, are challenged by the fact that results were substantially explained by demographic variables such as gender, age and education.

Our first major goal was to compare DM deficits under risk and ambiguity between SZ, AUD and HC. Both clinical groups showed deficits under risk, whereas only AUD also had impairments under ambiguity. In the GDT, both patient groups had significantly lower net scores than HC. Mean comparisons were solely significant within the nonrisky choices: SZ chose the second less risky option (three numbers) less frequently than HC. However, after controlling for the potential influence of demographics and IQ, the observed group differences were no longer statistically significant but still had a small to medium effect size. In agreement with earlier studies (Fond et al. 2013; Li et al. 2021; Pedersen et al. 2017; Zhang et al. 2015), our results suggest a weak but similar impairment in the reflective system in SZ and AUD.

Regarding DM under ambiguity, SZ showed no impairments within the first four blocks of the IGT but differed on a trend level from the performance of AUD. The main group difference was observed between AUD and HC; specifically, AUD exhibited a significant propensity towards making more disadvantageous choices from Deck B and chose significantly less often from Deck C than SZ. The DM behaviour of AUD in the IGT confirms a tendency to prefer short‐term rewards from risky decks while neglecting long‐term negative consequences. This could indicate hyperactivity of the impulsive system coupled with reduced activity of the reflective system, as described by Bechara (2005). SZ did not show a preference for disadvantageous choices (Decks A and B), which is in contrast to the majority of previous studies (Betz et al. 2019; Fond et al. 2013; Lee et al. 2007; Pedersen et al. 2017; Shurman et al. 2005; Xu et al. 2021; Zhang et al. 2015) and could suggest an impaired reflective, but not impulsive system.

Further, blockwise analyses of the IGT revealed a main effect of block and a main effect of group. An additional trend for an interaction resulted from descriptively fewer differences between the groups in the first two blocks, a slower increase in performance in AUD and a decline in performance of SZ in the fifth block. Significant differences were observed between SZ and HC versus AUD in the third block, as well as between AUD and HC in the fourth block. Even though the difference between SZ and HC was not significant in the last block and therefore must be interpreted with caution, this finding is in line with previous studies (Fond et al. 2013; Lee et al. 2007; Li et al. 2021) and might be explained by the change of the IGT in the course of the trials from ambiguity to risk (Brand, Grabenhorst, et al. 2007; Brand, Recknor, et al. 2007). This finding further suggests impairments in the reflective system only in SZ. The increasingly better performance of AUD across the IGT contrasts with evidence from a meta‐analysis that showed continuingly impaired performance in AUD (Ariesen et al. 2023). However, group differences were no longer significant after controlling for demographics.

The second major goal targeted the impact of EF (indicated by n‐back performance) on DM in SZ. Indeed, both SZ and AUD performed worse in the n‐back task compared to HC with moderate to large effect size (*η* ^2^ = 0.082), which is consistent with other reports of EF deficits in SZ (Fett et al. 2020; Forbes et al. 2009; Lee and Park 2005; Lett et al. 2014) and in AUD (Goldstein et al. 2004; Pitel et al. 2007; Ramey and Regier 2019). In line with previous studies (Brand et al. 2006; Brand, Recknor, et al. 2007; Fond et al. 2013), our results showed that higher GDT net scores among HC are strongly correlated with higher scores in the EF task. The moderate association in SZ is in line with the findings of Fond et al. (2013) and Zhang et al. (2015). In contrast, Lee et al. (2007) showed no association between EF and GDT performances in SZ (Lee et al. 2007). The lack of relationship between the GDT net score and EF in AUD suggests a specific impact of EF on DM in SZ. All in all, our results do not clearly confirm the proposed association between EF, respectively, working memory, and DM under risk. Thus, in line with Brand et al. (2005), we conclude that GDT performance is rather mediated by other EF than working memory in AUD and probably also in SZ (e.g., planning and shifting).

The positive association between IGT net scores and working memory was specific to SZ and not observed within the other groups. This suggests a closer association of working memory impairments with DM under ambiguity in SZ than with DM under risk. This highlights the importance of cognitive functions, in particular working memory, for advantageous DM performance under ambiguity (Bagneux et al. 2013; Betz et al. 2019; Woodrow et al. 2019). In general, our findings suggest a major problem in the reflective system in SZ with a prominent role of EF in DM and thus suggest a specific impairment profile in SZ. However, the impairments in the IGT in AUD, but not in SZ, do not rule out deficits in the impulsive system in SZ but rather suggest that these deficits are more marked in AUD. Future studies should go beyond the correlational design and train cognitive processes related more to the reflective system (e.g., working memory, planning and logical reasoning) versus the impulsive system (e.g., response inhibition, feedback processing and emotion regulation) in SZ to experimentally explore the impact of the reflective versus impulsive system on DM in SZ. In addition, studies are needed that further explore the role of the dopaminergic system and reward processing on DM in SZ. Although deficits in the GDT are theoretically clearly linked to an affected reflective system, they could also be influenced by impaired reward prediction error and loss devaluation (Verharen et al. 2018). In addition, we found a tendency for a moderate negative association between the PANSS positive subscale and the IGT net score and a tendency for a correlation of the PANSS positive subscale and the working memory performance. Neither GDT performance was associated with positive psychopathology nor the other PANSS subscales with DM or working memory performance. These results are partly in line with previous studies, which found no association between DM and symptom severity in SZ (Li et al. 2021) but are in contrast to Larquet et al. (2010), who showed differences depending on positive and negative symptoms. Regarding the antipsychotic medication, we found no association, neither to DM nor to working memory performances. This accords with the majority of earlier studies, which stated no influence of antipsychotic medication (Fond et al. 2013; Pedersen et al. 2017; Struglia et al. 2011) but is in contrast to the findings of Beninger et al. (2003).

Impaired DM has been observed in both inpatient and outpatient settings, as well as in younger and middle‐aged SZ (Fond et al. 2013; Li et al. 2021; Pedersen et al. 2017), suggesting that it occurs independently of the course of the disease and the treatment setting. Unfortunately, we have no valid assessment of the duration of illness or the timing of diagnosis in our study. Although previous studies (Fond et al. 2013; Lee et al. 2007; Li et al. 2021; Pedersen et al. 2017) found no link between IGT/GDT performance and illness duration, future research is needed for confirmation. The substantial impact of demographic variables on the reported results constitutes a major limitation for clear conclusions. Group differences in gender, age and education between the inpatient samples, especially, could not be compensated in the present sample selection. After including the covariates, the results were no longer significant and had reduced effect sizes. Notwithstanding a possibly reduced power due to the addition of covariates into the ANOVAs (Bernerth and Aguinis 2016), our findings suggest a strong influence of demographic variables on DM under ambiguity and under risk. In addition, our SZ sample was heterogeneous, and an issue that was not addressed in our study is whether depression and anxiety influenced DM. Because depressive mood seems to negatively bias the perception and interpretation of ambiguous cues (Lin et al. 2019), this could also transfer to DM under ambiguity. A review by de Siqueira et al. (2018) showed that depressive patients also exhibit impaired DM and tend to make more disadvantageous decisions compared to HC. Thus, future studies on DM in SZ should consider comorbid depressive and anxiety symptoms and may differentiate between SZ and patients with schizoaffective disorders. An additional limitation is the fixed task order that might partially explain the decline in the fifth block of the IGT within SZ. Further research could vary the order of tasks to better control for potential order effects and fatigue and could apply an IGT without deck limits to examine what might explain the performance drop within SZ. Applying an IGT without deck limits can show whether the drop in the fifth block is related to EF deficits that impair performance when the task switches from ambiguity to risk, or to inherent deficits in adjusting to new rules or new optimal choices in SZ due to impaired probabilistic learning (Reddy et al. 2016). Furthermore, the duration of the tasks could also have an impact on the performance, as the completion of the IGT took significantly longer than the GDT. The well‐known impairments in attentional control and working memory in SZ (Forbes et al. 2009) could affect performance in DM tasks by leading to concentration deficits as task duration increases. Thus, it is an open question whether performance in the GDT would also decrease in SZ if the task were longer. Future studies may apply a GDT with more trials for examining potential further performance decreases within the GDT due to concentration deficits.

## 5 Conclusion

Taken together, the present study offers new evidence on DM characteristics in SZ, documenting a differential DM profile in SZ and AUD, and a specific impact of EF on DM under ambiguity in SZ. Both clinical samples showed a propensity for impairments in the reflective system, but the impulsive system seems only affected in AUD. Both groups could benefit from EF training, particularly targeting planning, categorization and cognitive flexibility. AUD may additionally benefit from interventions focused on impulse control and response inhibition (Verdejo‐Garcia et al. 2018) to improve DM. As a next step, and after confirming the robustness of our results by strict consideration of demographic covariates, the association of cognitive processes related to the reflective versus impulsive system in SZ might be probed by experimental variation (e.g., training one or the other variable).

## Author Contributions

**Sarah Stumpp:** data curation, formal analysis, investigation, methodology, project administration, visualization, writing – original draft. **Alexander Wolber:** data curation, investigation, project administration, writing – review and editing. **Natascha Büchele:** data curation, investigation, project administration, writing – review and editing. **Leonie Lipinski:** data curation, investigation, project administration, writing – review and editing. **Stephanie N. L. Schmidt:** data curation, investigation, methodology, project administration, software, writing – review and editing; **Brigitte Rockstroh:** conceptualization, funding acquisition, methodology, resources, supervision, writing – review and editing. **Michael Odenwald:** conceptualization, funding acquisition, methodology, resources, writing – review and editing. **Daniela Mier:** conceptualization, funding acquisition, methodology, resources, supervision, writing – review and editing.

## Funding

This work was supported by Deutsche Forschungsgemeinschaft (MI 1975/7‐1 and OD 113/2‐1).

## Conflicts of Interest

The authors declare no conflicts of interest.

## Data Availability

The data supporting the findings of this study are available at https://osf.io/7kagr/files/osfstorage, with the exception of data from two participants in the schizophrenia group who did not provide informed consent for public data sharing.
